# Systematic Characterisation of the Fragmentation of Flavonoids Using High-Resolution Accurate Mass Electrospray Tandem Mass Spectrometry

**DOI:** 10.3390/molecules29225246

**Published:** 2024-11-06

**Authors:** Candy Jiang, Paul J. Gates

**Affiliations:** School of Chemistry, University of Bristol, Cantock’s Close, Bristol BS8 1TS, UK

**Keywords:** flavonoids, mass spectrometry, fragmentation, structure characterisation

## Abstract

Flavonoids are a class of polyphenolic secondary metabolites found in plants. Due to their ubiquity in our daily dietary intake and their major anti-oxidative, anti-inflammatory and anti-mutagenic activities, they have been a major focus of wide-ranging research for the past two decades. Mass spectrometry combined with liquid chromatography is one of the most popular techniques for the analysis of flavonoids. In this study, high-resolution accurate mass electrospray tandem mass spectrometry was used to study 30 flavonoids in both positive and negative ionisation modes. From the data obtained, common losses were summarised and compiled. Dominating neutral losses were tabulated. The radical loss of CH_3_· was observed in flavonoids containing methoxy groups and three key diagnostic product ions were identified. These were *m*/*z* 153 (indicative of two OH groups on ring A) *m*/*z* 167 (indicative of one OH and one methoxy group on ring A) and *m*/*z* 151 (a flavanol, with no ketone oxygen but two OH groups on ring A). These will be useful in structural elucidation of unknown flavonoids and flavonoid metabolites. Energy breakdown graphs were utilised to distinguish between three pairs of structural isomers, and to help rationalise proposed fragmentation pathways. Lastly, a competition of loss of CH_3_· and methane was reported for rhamnetin and isorhamnetin in the negative ion mode for the first time. Proposed fragmentation pathways were given to rationalise the differences in peak intensities for this competitive process.

## 1. Introduction

Flavonoids are a class of polyphenolic secondary metabolites mainly found in plants [1]. They are present in fruits, nuts and vegetables and have become a part of our daily dietary intake. Flavonoids often contribute to the colour of flowers, where they assist in pollination and provide UV protection [2]. Plants use them to aid growth and as a defence against disease and infection [2] and, due to their unique structures, flavonoids have been found to have many important biological activities such as anti-oxidative, anti-inflammatory, anti-mutagenic and anti-carcinogenic [3,4,5].

For example, the isoflavonoid glabridin from *Glycyrrhiza glabra* has been found to inhibit low-density lipoprotein oxidation by scavenging free radicals [6]. The human metabolism of flavonoids has been studied and reviewed in detail [7], along with the mechanism for their anti-oxidant activity, absorption and bioavailability [8,9,10]. It has been noted that the configuration and number of hydroxyl groups and the substitution of the various functional groups on the structures of individual flavonoids affect their bioavailability, metabolism and biological activities [11]. Due to their diverse range of chemical and biological properties, their extensive applications include nutraceutical, pharmaceutical, medical and cosmetic uses [12,13].

Increasing awareness of the health benefits and pharmaceutical applications of flavonoids has led to a considerable increase in the number of studies of flavonoids using mass spectrometry (MS). The amount of the available literature on flavonoid analysis by various MS techniques can be overwhelming. The earlier studies of flavonoids were performed using electron ionisation (EI), methane chemical ionisation (CI) and fast atom bombardment (FAB) MS [14,15,16,17]. Results stated that the outcome of the spectra was heavily dependent on the selection of the MS technique. Peak intensities of specific ions in the resulting mass spectra depended on the ionisation technique and the structure of the analytes.

As the application of electrospray ionisation (ESI) became mainstream, studies started to implement ESI coupled with high-performance liquid chromatography–tandem mass spectrometry (HPLC-MS/MS) to investigate various classes of flavonoids and their substitutes [18,19]. Recent advances in the analytical approach for the study of flavonoids using ESI and other modern MS techniques have been readily and widely reviewed [20,21,22]. Other analytical methods looked into coupling unified chromatography and ultra-performance liquid chromatography with ESI to look into various flavonoid groups [23,24]. Direct analysis in real time (DART) coupled with MS is also employed to look into flavonoid content in bee products for food quality purposes [25].

From the MS/MS studies of flavonoids, specific fragmentations such as dehydration and loss of CO from protonated molecular ions were predominantly found [17]. The fragmentation of flavonoids was explored in the negative ion mode [26,27]. Several fragmentation pathways were proposed based on highly specific negative product ions due to the retro Diels–Alder (RDA) process. Results from these studies demonstrated that, in the analysis of flavonoids, the positive ion mode gives more structural information, but, when combined with the negative ion mode, could provide sufficient information for identifying unknown flavonoids compounds and new metabolites without resorting to challenging purification methods [28,29].

One common challenge in the analysis of flavonoids is the vast number of variations in their functional group’s position, mainly in the locations of the hydroxy and the methoxy groups on the side rings. This results in a dominating presence of structural isomers within the same flavonoid subclasses. Since all MS techniques are based on mass-to-charge ratio (*m*/*z*) separation, separating these structural isomers is challenging. Errors resulting from unreliable peak separation or spectra interpretation are common. Therefore, after examining the existing literature, this study selected 30 commercially available flavonoids from 6 flavonoid classes and performed a systematic MS/MS analysis in both the positive and negative ion modes using ultra-high-resolution accurate mass ESI-MS/MS.

This systematic approach enabled the identification of three key diagnostic product ions, which can be used as fingerprints to investigate unknown flavonoids. Integration of the positive and negative ion modes provided complementary data. Energy breakdown graphs were sketched to demonstrate the relationship between collision energy and peak intensity of the product ions of selected isomeric flavonoid pairs, and to display distinctive features for each flavonoid that would otherwise be overlooked. Complete fragmentation pathways were also proposed for selected flavonoids. This paper also investigated the competition between neutral loss and radical loss observed for rhamnetin and isorhamnetin due to the position variation of the methoxy moiety.

## 2. Results and Discussion

**Figure 1:**
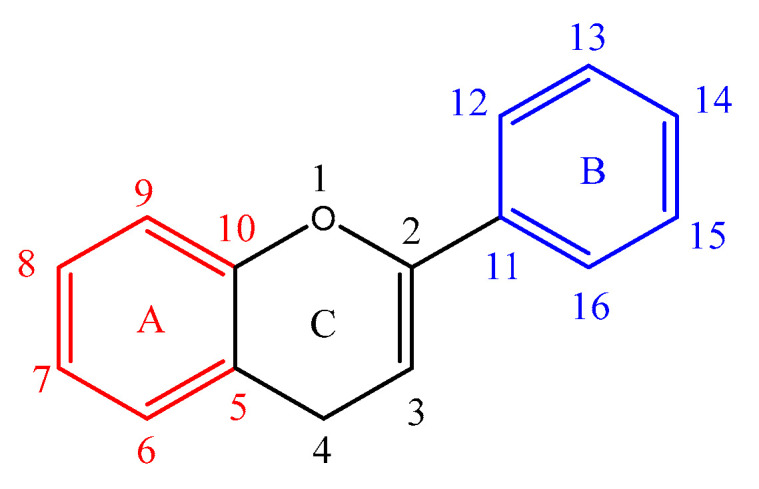
The basic structure of flavonoids. The rings are labelled A to C and the atom numbering run from 1–16. This system is used throughout the study.

Based on the previous literature, [30] a numbering system is adapted in this paper. Figure 1 shows the basic structure of a flavonoid. Ring A is marked in red, ring B in blue and ring C in black. The atom numbering starts from the heterocyclic oxygen in ring C and ends on the ring B benzene carbon. A nomenclature is also added to clarify the assignment of the product ions. For example, a ring cleavage labelled ‘**rC^x,y^_AorB_**’ represents the cleavage location on ring C between carbon–carbon positions x and y, leaving either ring A or B intact.

### 2.1. Common Losses in the Positive Ion Mode

Positive ion mode ESI-MS/MS spectra are collected for all 30 flavonoids, and the neutral/radical losses from the precursor ions are tabulated (see Table A2 in Appendix A). The first trend to note is that flavonoids from the same group do not always have the same common losses, although there are degrees of similarity. Secondly, common losses are mostly dependent on the location and number of –OH groups (loss of H_2_O), –MeO groups (loss of CH_3_ radical) and the presence of a C=O function in ring C (loss of CO, mass 28). Any losses higher than 100 mass units result from ring cleavage through the RDA processes on ring C. Table 1 summarises the typical neutral losses observed in the positive ion mode.

From the common losses, three key diagnostic product ions are found to be directly related to the structure of their precursor ion (PI). These are *m*/*z* 167, *m*/*z* 153 and *m*/*z* 151. Among these, *m*/*z* 153 is the most commonly observed and results from cleavage on ring C between atoms 1 and 4, leaving ring A intact, and is therefore designated as rC^1,4^_A_. Product ion *m*/*z* 167 is also due to cleavage on ring C between atoms 1 and 4, leaving ring A intact, and is therefore designated as rC^1,4^_A_. The difference between these two ions is due to a methoxy group on ring A. Lastly, ion *m*/*z* 151 is formed from cleavage on ring C between carbons 3 and 10, therefore designated as rC^3,10^_A_. All three product ions are listed in Table 2, along with the flavonoids (and their classes) that produced them.

Table 3 is a demonstration of the three key diagnostic properties of the product ions allowing for the observation of the structural correlation between precursor and product ions. Quercetin, sakuranetin and epigallocatechin are used as examples. All three product ions are coloured to highlight the exact structural resemblance to their respective PIs. Therefore, it can be concluded that the presence of *m*/*z* 153 demonstrates the presence of two OH groups in ring A (at C-6 and 8) and a C=O at C-4 in ring C. Observation of *m*/*z* 167 indicates the presence of an OH group at C-6, a methoxy group at C-8 and a ketone at C-4 on ring C. Finally, product ion *m*/*z* 151 shows the presence of no ketone on ring C i.e., C-4 is an unoxidised CH_2_, but two OH groups on ring A at C-6 and C-8. The presence of these product ions gives a clear insight into the structures of their respective flavonoid precursor ions. These findings will be useful in the structural elucidation of unknown flavonoids.

### 2.2. Energy Breakdown Graphs for Isomeric Differentiation

Energy breakdown graphs (EBGs) are plots of collision energy versus relative peak intensity for the selected product ions. They can demonstrate the different energy thresholds for alternative fragmentation routes and showcase secondary losses during the fragmentation processes. Because of this, they have been used previously for a variety of MS^n^ studies as a complementary analytical tool to provide additional information of the fragmentation processes [31,32,33]. EBGs have previously been used in the study of flavonoids to examine how changes in collision energy affect peak intensities for selected product ions [34]. Due to the unique ability of EBGs to display a complete picture of the relationship between product ion peak intensities vs. collision energy, they have also been employed for isomer differentiation [35,36,37,38]. Product ions of a pair of structural isomers can often exhibit different peak intensities under the changes in collision energy. The same pair of structural isomers could also generate unique product ions as the collision energy increases. These specific characteristics can all be captured and visualised by EBGs. Therefore, EBGs are a powerful tool in addition to MS/MS spectra. In this part of the paper, EBGs are plotted for three pairs of isomeric flavonoids from their MS/MS spectra in the positive ion mode.

#### 2.2.1. Rhamnetin and Isorhamnetin

**Figure 2:**
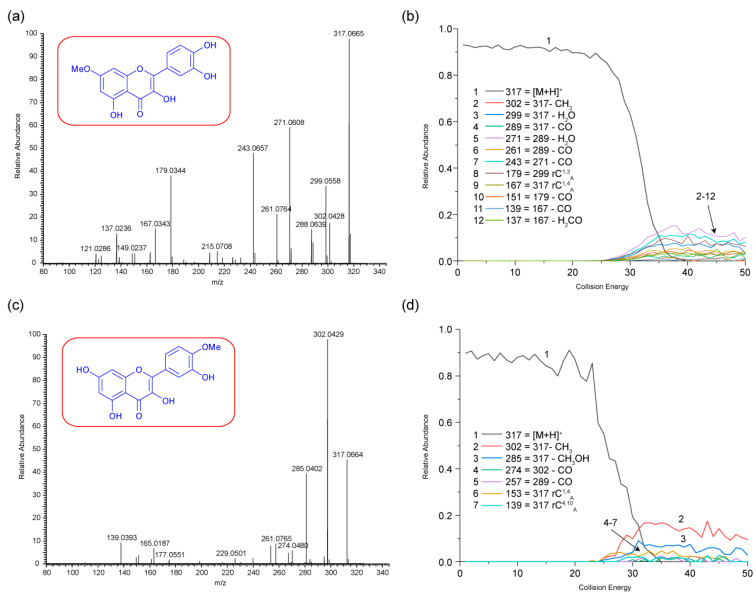
The positive ion mode CID MS/MS spectra and energy breakdown graphs (EBGs) of rhamnetin and isorhamnetin. Spectrum (**a**) is the MS/MS (32 eV and PI *m*/*z* 317) and graph (**b**) is the EBG for rhamnetin, whereas spectrum (**c**) is the MS/MS (31 eV and PI *m*/*z* 317) and graph (**d**) is the EBG isorhamnetin.

Rhamnetin and isorhamnetin are structural isomers differentiated by the location of the methoxy group (see Figure 2 for structures). Each of them has one methoxy group and four OH groups. Their energy breakdown graphs shown in Figure 2 indicates a few differences. Firstly, there are fewer product ions for isorhamnetin. Secondly, the difference in the intensity of product ion *m*/*z* 302 is almost four times more intense for isorhamnetin compared with rhamnetin. These observations are both due to the intensity of the loss of the methyl radical. In the case of rhamnetin, the losses of H_2_O and CO compete and are a much preferred fragmentation route. Except for the common losses of the methyl radical and of a CO, rhamnetin and isorhamnetin have noticeably different fragmentation pathways despite being structural isomers.

**Scheme 1:**
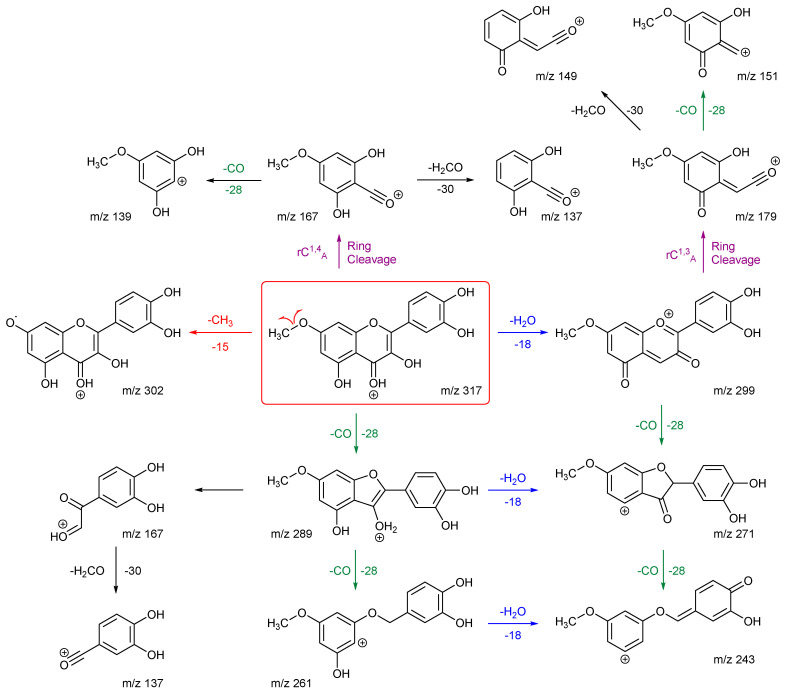
The proposed fragmentation pathway for rhamnetin in positive ion mode. The formulae of all product ions are confirmed by accurate mass (2.4 ppm) (Table A4 in Appendix B).

**Scheme 2:**
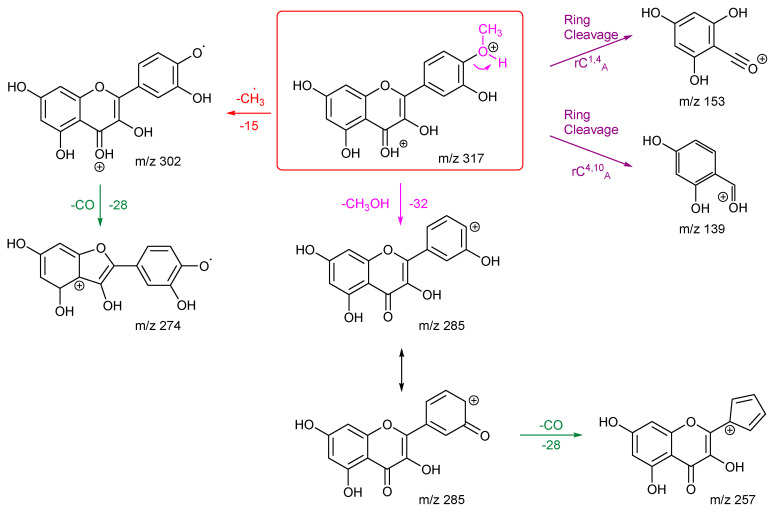
Proposed fragmentation pathway for isorhamnetin in positive ion mode. The formulae of all product ions are confirmed by accurate mass (average of 2.7 ppm) (Table A5 in Appendix B).

The fragmentation pathways and proposed structures of the product ions observed in the analyses of rhamnetin and isorhamnetin are shown in Scheme 1 and Scheme 2. The protonation site is on the carbonyl oxygen of ring C unless stated otherwise. Scheme 1 shows multiple losses of CO from rhamnetin, either after an initial loss of H_2_O or directly from the precursor ion. The competition between the loss of a methyl radical and other routes makes this route less favourable. The two ring cleavages are marked in purple. The first of which occurs after a water loss to produce *m*/*z* 179, which is the third most intense peak in the EBG. The second ring cleavage results in *m*/*z* 167. However, this peak can also be produced through a loss of CO along with a C–O bond cleavage. Both routes go on to further lose H_2_CO to produce *m*/*z* 137, with the structure at the bottom left of the scheme being proposed as being the most likely.

Isorhamnetin has fewer product ions than rhamnetin, with *m*/*z* 302 as the most prominent. This can be explained by the formation of a stabilised radical, the structure of which is shown in Scheme 2. Compared with the structure of ion *m*/*z* 302 for rhamnetin, *m*/*z* 302 for isorhamnetin is stabilised by the adjacent hydroxy group and conjugation through ring B and C. The production of *m*/*z* 274 further proves this, as product ion *m*/*z* 302 is stable enough to undergo secondary fragmentation by loss of CO. A ring closure is proposed for ion *m*/*z* 274 to explain why it does not fragment further. The second most intense peak is *m*/*z* 285, which is the result of the loss of methanol. Protonation is proposed to be on the methoxy oxygen to induce an ortho elimination. This specific loss for isorhamnetin is indicative of the presence of a methoxy group on ring B at the ortho position, agreeing with the previous literature [34]. Kaempferide, which has a methoxy group at the same location, exhibits the exact same mechanism [39].

The structural differences between rhamnetin and isorhamnetin also affect the location of ring cleavages. Isorhamnetin has two ring cleavages: _r_C^4,10^_A_ and _r_C^1,4^_A_. This is very different to rhamnetin, and it shows that, even though both flavonols have the same number of OH groups and a methoxy group, the location of these functional groups significantly affects the formation of the product ions. The relative stability of the different radical ions produced influences the entire spectra. The stability of the ring B product ions also influences which fragmentation pathways would be followed.

#### 2.2.2. Kaempferol and Fisetin

**Figure 3:**
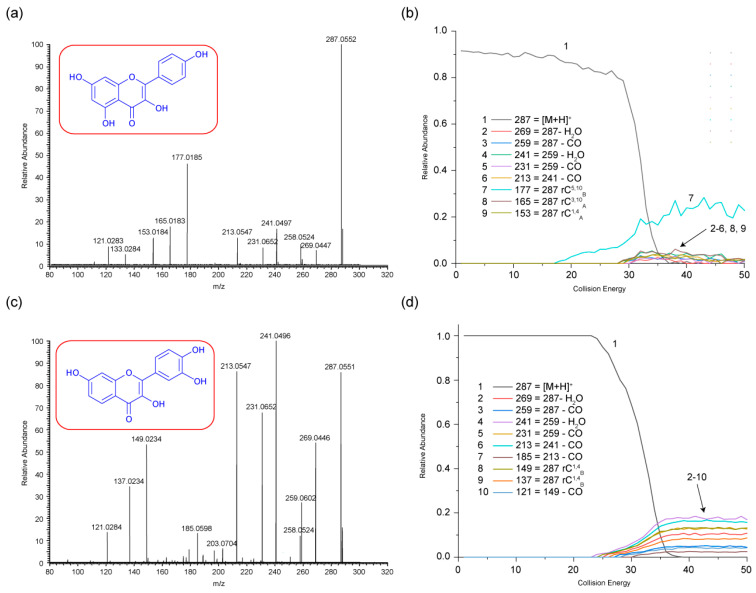
The positive ion mode CID MS/MS spectra and energy breakdown graphs (EBGs) of kaempferol and fisetin. Spectrum (**a**) is the MS/MS (31 eV and PI *m*/*z* 287) and graph (**b**) is the EBG for kaempferol, whereas spectrum (**c**) is the MS/MS (32 eV and PI *m*/*z* 287) and graph (**d**) is the EBG fisetin.

As with rhamnetin and isorhamnetin, both kaempferol and fisetin belong to the flavonol class. Both kaempferol and fisetin have four OH groups in their structures. However, kaempferol only has one OH group on ring A, whereas fisetin has two. The converse is true for ring B (see structures in Figure 3). Differences in their product ions can be viewed in their energy breakdown graphs. Kaempferol has a dominant ion at *m*/*z* 177, resulting from a ring cleavage, whereas fisetin favours the loss of mass 46 to produce the ion at *m*/*z* 241. Product ion *m*/*z* 177 occurs at a much lower collision energy than the others for kaempferol. All product ions of fisetin occur at a collision energy of around 23 eV, as seen in the energy breakdown graph.

**Scheme 3:**
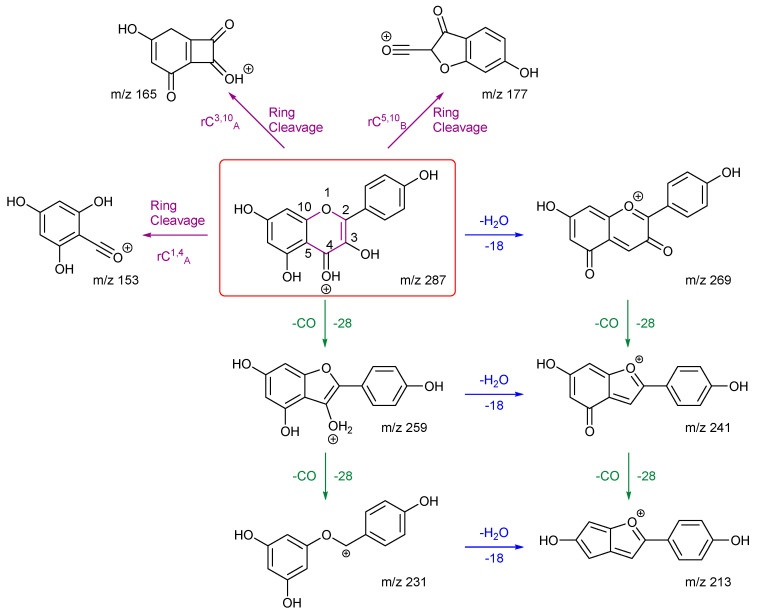
Proposed fragmentation pathway for kaempferol in positive ion mode. The formulae of all product ions are confirmed by accurate mass (average of 0.8 ppm) (Table A6 in Appendix B).

**Scheme 4:**
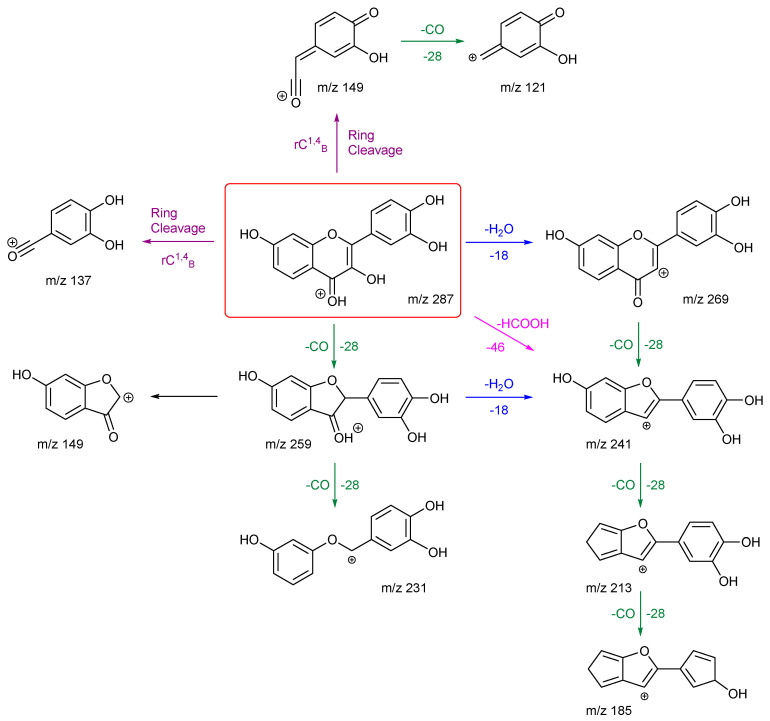
Proposed fragmentation pathway for fisetin in positive ion mode. The formulae of all product ions formed are confirmed by accurate mass (average of 0.4 ppm) (Table A7 in Appendix B).

The fragmentation pathways and proposed structures of the product ions observed in the analyses of kaempferol and fisetin are shown in Scheme 3 and Scheme 4. The following discussion aims to rationalise the proposed product ion structures and their EBGs. Kaempferol has extensive ring cleavages, and the bonds broken are coloured in purple. The high peak intensity of product ion *m*/*z* 177 could be due to a stabilised five-membered ring structure with a tertiary carbocation. The key diagnostic product ion *m*/*z* 153 is present with kaempferol. Extensive H_2_O and CO losses are also observed.

Fisetin follows the same number of H_2_O and CO losses to produce product ions *m*/*z* 269, 259, 241, 231 and 213. From its EBG, the ion at *m*/*z* 241 is the most intense and occurs at much lower energy than the others. This suggests that it may be due to the loss of formic acid (HCOOH) in one step, as highlighted in Scheme 4, in addition to the first loss of H_2_O, followed by a CO. It then goes on to lose a CO to produce the ion at *m*/*z* 213. This agrees with the observation that the ion *m*/*z* 213 is the second most intense peak in the EBG. Compared with kaempferol, it has one less ring cleavage, and both product ions from these cleavages leave ring B intact. Product ion *m*/*z* 149 has two fragmentation pathways, with one occurring directly from the cleavage of ring C at atoms 1 and 4, the other is by a carbon–carbon bond breakage from ion *m*/*z* 259. The first is more likely according to the EBG. Both routes can be followed by another loss of CO to give product ion *m*/*z* 121. Finally, the ion at *m*/*z* 185 has the lowest intensity and requires the highest collision energy. This can be explained by a less favourable charge remote fragmentation and the need to break the already stabilised ring structure of *m*/*z* 213.

Kaempferol and fisetin differ in their locations, numbers and remaining structures of the ring C cleavages. Product ions of these ring cleavages are key to their structural identification. Kaempferol has a key diagnostic product ion *m*/*z* 153, which can distinguish it from fisetin due to the location of the OH group.

#### 2.2.3. Chrysin and Daidzein

**Figure 4:**
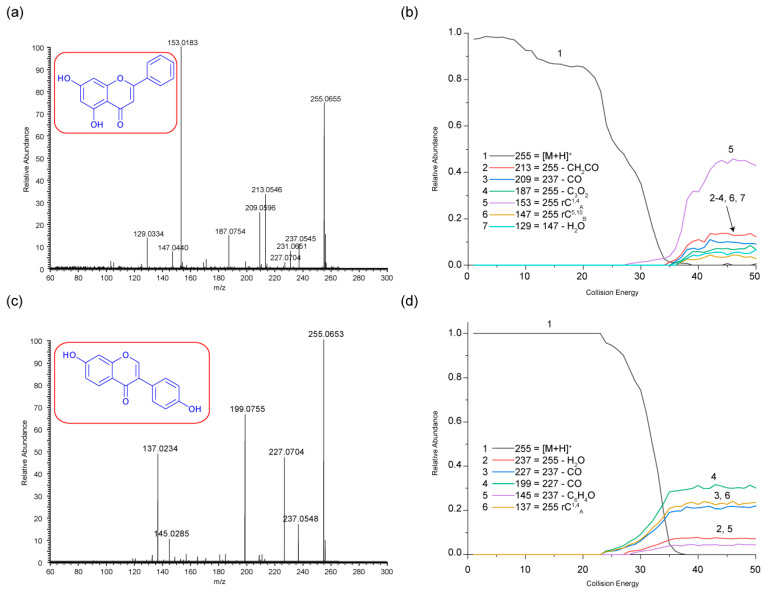
The positive ion mode CID MS/MS spectra and energy breakdown graphs (EBGs) of chrysin and daidzein. Spectrum (**a**) is the MS/MS (35 eV and PI *m*/*z* 255) and graph (**b**) is the EBG for chrysin, whereas spectrum (**c**) is the MS/MS (30 eV and PI *m*/*z* 255) and graph (**d**) is the EBG daidzein.

The final pair of isomeric flavonoids are chrysin and daidzein. They are selected as they are structural isomers but belong to different flavonoid classes. Both of their protonated molecular ions occur at *m*/*z* of 255. Their highly distinctive EBGs are presented in Figure 4. The most intense peak for chrysin is the product ion *m*/*z* 153, the key diagnostic peak from a specific structure on ring A. This peak also first appears at a lower collision energy compared with others. In contrast, daidzein has three product ions at the lower collision energy. These are *m*/*z* 199, 137 and 227.

**Scheme 5:**
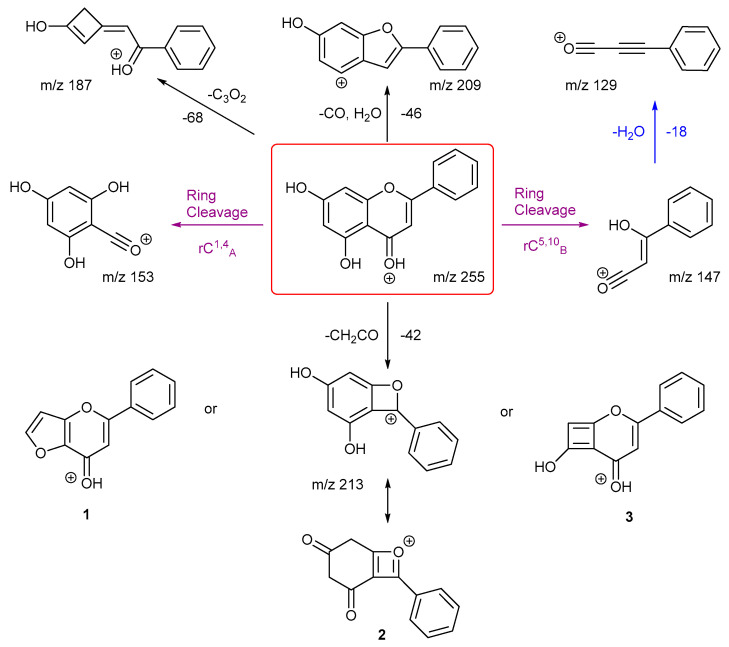
Proposed fragmentation pathway for chrysin in positive ion mode. The formulae of all product ions formed are confirmed by accurate mass (average of 0.5 ppm) (Table A8 in Appendix B).

**Scheme 6:**
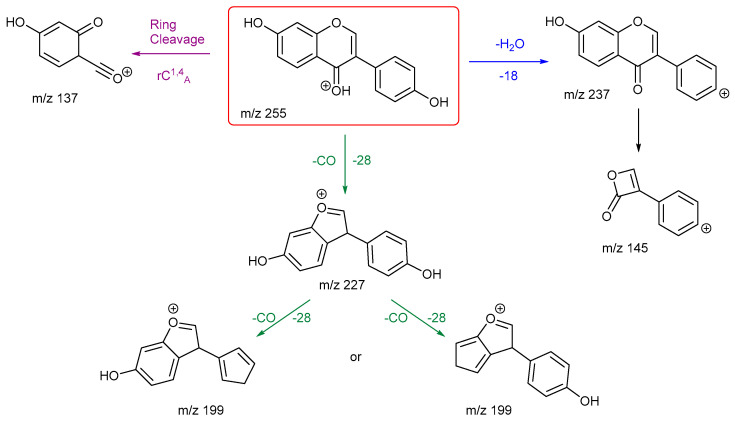
Proposed fragmentation pathway for daidzein in positive ion mode. The formulae of all product ions formed are confirmed by accurate mass (average of 0.6 ppm) (Table A9 in Appendix B).

The proposed fragmentation scheme for chrysin and daidzein are shown in Scheme 5 and Scheme 6. Chrysin has two ring cleavages, including the key diagnostic product ion for ring structure A. Also, it has a loss of formic acid to produce ion at *m*/*z* 209. There are also extensive ring contractions for chrysin, which agrees with the previous literature [40]. The product ion *m*/*z* 187 is created from a very unusual loss of carbon suboxide (C_3_O_2_,), first observed in 2001 for flavones and later in a few other studies both in positive and negative ion mode [40,41,42]. Although no mechanisms have been proposed. This could be an opportunity for future work to investigate a viable mechanism. Also, a loss of CH_2_CO gives three possible structures for product ion *m*/*z* 213. Structure **3** has been shown previously in the literature [40]. However, we believe structure **1** is more stabilised.

Daidzein has only one ring cleavage from the protonated PI, which results in product ion *m*/*z* 137. The most intense peak *m*/*z* 199 is produced from the loss of a CO from *m*/*z* 227. Two possible structures are proposed for this ion; both involve a ring contraction to form a cyclopentadiene in their structure, depending on which CO is lost. Product ion *m*/*z* 145 has the lowest intensity, possibly due to a less favourable charge remote ring cleavage from ion *m*/*z* 237. In summary, for both chrysin and daidzein, ring cleavage on ring C is very energetically favourable. Chrysin has extensive ring contractions that result in the unusual neutral loss of C_3_O_2_. Daidzein can also undergo ring contraction to lose a CO.

### 2.3. Common Losses in the Negative Ion Mode

MS/MS data for the 30 flavonoids in negative ion mode are also investigated, and neutral/radical losses of their deprotonated precursor ions are summarised in Appendix A Table A3. Flavonoids are grouped and marked by different colours based on their classes, as in the positive ion mode. The first trend is consistent with the observations in the positive ion mode. Flavonoids from the same classes do not always exhibit the same common losses. They only have the same common losses if they are structural isomers from the same flavonoid class. For example, in the flavone group, genkwanin and diosmetin both only produce a loss of 15, which corresponds to a CH_3_· radical loss. Gallocatechin and epigallocatechin, and catechin and epicatechin, are two pairs of structural isomers that are all flavanols; hence, they have the same neutral losses.

Secondly, compared with the positive ion mode CID MS/MS data, there are more lower mass neutral losses. The most common ones are summarised in Table 4. Like with the positive ion mode, common losses in the negative ion mode are also dependent on the hydroxy and methoxy group locations. Other than the predominantly occurring loss of H_2_O, loss of CO is frequently observed. Any losses with higher than 100 mass units is likely to be an indication of ring cleavage on the ring C.

Results from the negative ion mode MS/MS have one major difference from the positive ion mode spectra: the loss of 16, which corresponds to a CH_4_ methane loss. This has been previously published in the literature of this research group [43], and the data acquired here are strong evidence to further support this observation. Flavonols with a neutral loss of CH_4_ are highlighted in the box in Table A3. These are discussed and investigated further using energy breakdown graphs and proposed mechanisms.

### 2.4. Loss of CH_3_· and CH_4_

Both rhamnetin and isorhamnetin have a loss of 16, corresponding to the loss of methane confirmed by accurate mass measurement (see Table A10 and Table A11 in Appendix B). This observation of CH_4_ elimination is very interesting and has been published before with heterocyclic aromatic amines [44] and the flavanone hesperetin but not with any other flavonoids. Hence, it remains the focus of the last part of this paper.

Out of all the flavonoids with methoxy groups, only rhamnetin and isorhamnetin exhibit a loss of CH_4_ in their negative ion MS/MS spectra. However, all the flavonoids mentioned share a common loss of a CH_3_· methyl radical. This could be because the unique CH_4_ loss requires a hydroxyl group on the meta carbon in ring C, which the flavone groups lack. An OH group on the neighbouring carbon is also essential to facilitate this mechanism. This explains why, although kaempferide has a hydroxyl group on the meta carbon in ring C and a methoxy group on ring B, the lack of an OH group on the same ring prevents the loss of 16.

**Figure 5:**
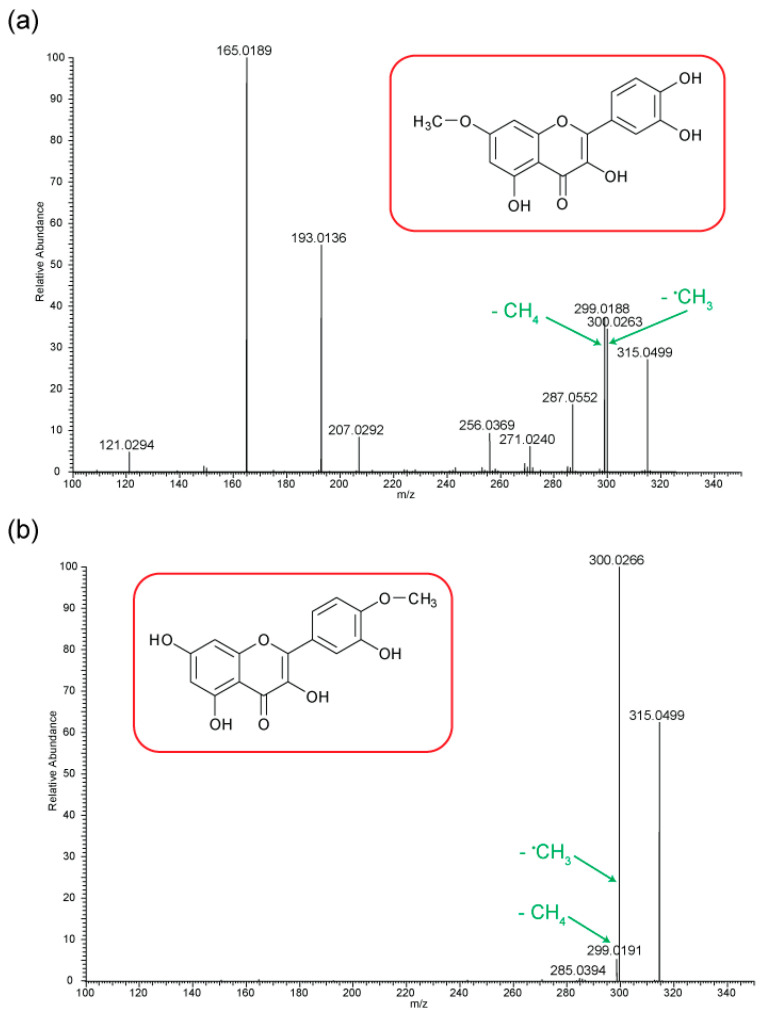
Negative ion ESI-MS/MS spectra of (**a**) rhamnetin and (**b**) isorhamnetin. PI *m*/*z* 315 in both cases. Product ions at *m*/*z* 300 (loss of CH_3_·) and *m*/*z* 299 (loss of CH_4_) are highlighted.

Figure 5 shows the negative ion MS/MS spectra for both rhamnetin and isorhamnetin. Although rhamnetin and isorhamnetin are structural isomers and belong to the same flavonol group, their negative ion MS/MS spectra are distinctively different. They both have losses of CH_3_· (*m*/*z* 300) and CH_4_ (*m*/*z* 299). Compared with isorhamnetin, *m*/*z* 299 of rhamnetin is at a much higher intensity and is slightly more intense than *m*/*z* 300. In contrast, for isorhamnetin, the loss of CH_3_· is a much more favourable route. For rhamnetin, a number of additional fragment routes are followed to generate losses of CO in much the same way as in positive ion mode. For isorhamnetin, the only additional product ion is *m*/*z* 285 (loss of MeOH from the PI), occurring at a very low intensity. The loss of the methyl radical totally dominates this spectrum.

**Figure 6:**
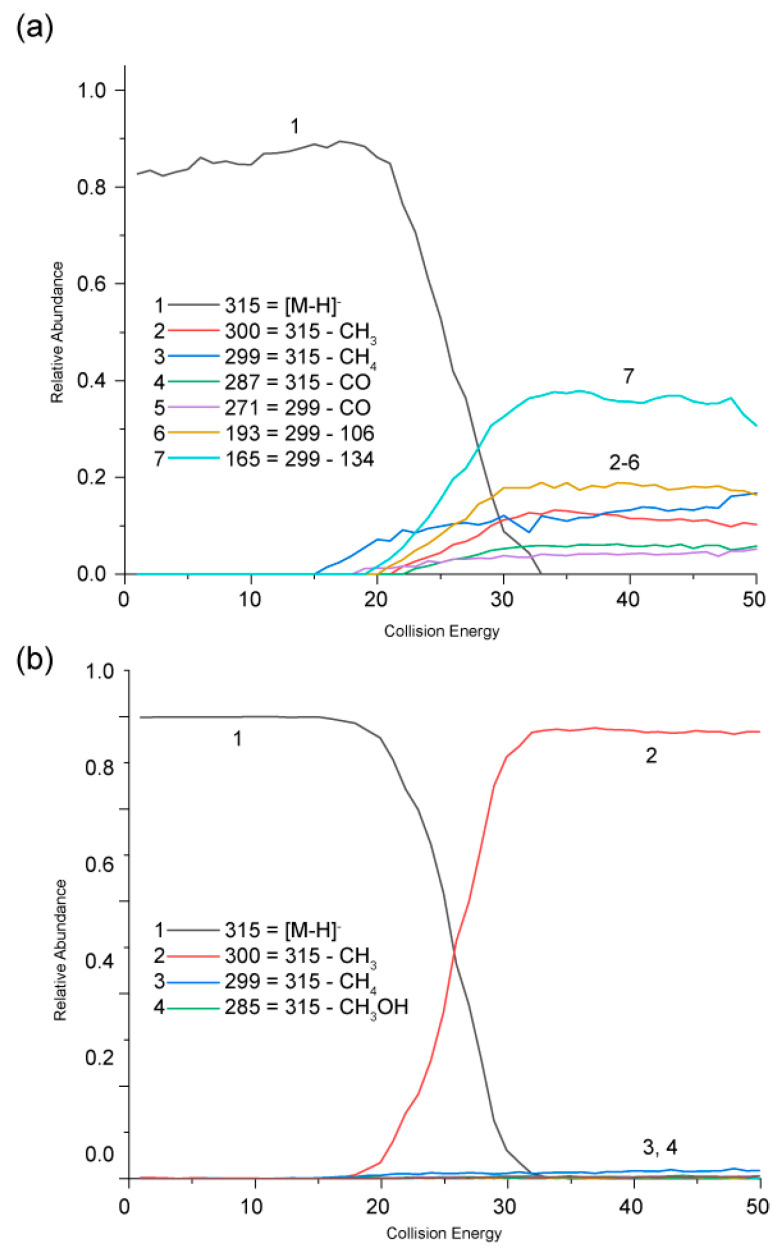
Energy breakdown graphs for (**a**) rhamnetin and (**b**) isorhamnetin for the negative ion ESI-MS/MS analysis.

Figure 6 shows distinctive differences in the product ions for these two flavonols despite the similarity in their structures. Rhamnetin produces more intense product ion peaks, especially in the lower mass range (*m*/*z* 165). Whereas the loss of CH_3_· dominates the isorhamnetin spectrum. In the breakdown graph for rhamnetin, peak *m*/*z* 299 starts to occur at CID energy 15eV and *m*/*z* 300 at just above 20 eV. As the collision energy increases, *m*/*z* 299 peak intensity also increases. This supports our theory that loss of CH_4_, in the case of rhamnetin, is more energetically favourable than the loss of CH_3_. Figure 6 shows very low intensity for the loss of CH_4_ for isorhamnetin and other product ions, except *m*/*z* 300. This observation could be supported by a different mechanism for the loss of CH_4_, which is discussed next. The striking differences in the mass spectra between these two isomeric flavonols in the negative ion mode build an excellent foundation for isomerisation differentiation. The unique loss of CH_4_ could be used for the structural elucidation of unknown flavonoids in future work.

**Scheme 7:**
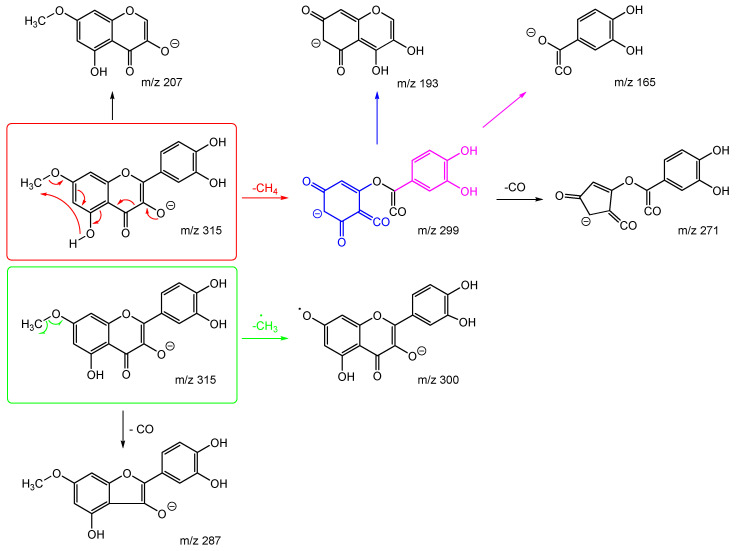
Proposed fragmentation pathway for rhamnetin in negative ion mode. The red route shows loss of CH_4_ and the green route shows loss of CH_3_. The formulae of all product ions are confirmed by accurate mass (average 2.6 ppm) (Table A10 Appendix B).

**Scheme 8:**
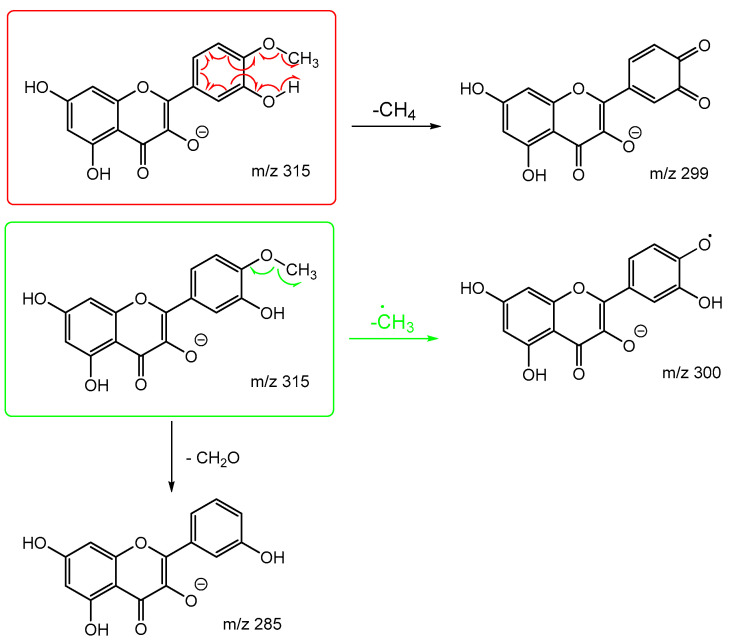
Proposed fragmentation pathway for isorhamnetin in negative ion mode. The red route shows loss of CH_4_ and the green route shows loss of CH_3_. The formulae of all product ions are confirmed by accurate mass (average 3.2 ppm) (Table A11 Appendix B).

Scheme 7 and Scheme 8 are the proposed fragmentation pathways for rhamnetin and isorhamnetin, including the proposed alternative mechanisms for the generation of product ions *m*/*z* 300 and 299 in the negative ion mode. In Scheme 7, the negative charge is proposed to be on the meta oxygen in ring C, facilitating a 1,5 hydride shift to eliminate CH_4_ on ring A. This also promotes a ring opening between C3 and C4 on ring C, resulting in the structure for product ion *m*/*z* 299 in Scheme 7. Fragmentation of *m*/*z* 299 further produces *m*/*z* 271, 193 and 165. For isorhamnetin, due to the different positions of the MeO and OH group, an alternative mechanism is proposed, as published in the literature for hesperetin [44]. Unlike the 1,5 hydride shift, this mechanism is less energetically favourable and could be a reason for the lower intensity of peak *m*/*z* 299 for isorhamnetin. The charge remote fragmentation and the lack of ring opening could also contribute to this lower intensity in Figure 5.

## 3. Materials and Methods

In this study [45], 30 flavonoids from 6 subclasses were analysed. They were all obtained from Sigma Aldrich (purity ≥ 90%). The flavonoids were dissolved in water/methanol (1:1, 1 mg/mL) to make up standard solutions. The flavonoids were quercetin, morin, rhamnetin, isorhamnetin, kaempferide, diosmetin, kaempferol, fisetin, 5-hydroxyflavone, galangin, baicalein, apigenin, luteolin, scutellarein, chrysin, daidzein, 6-hydroxyflavanone, 7-hydroxyflavonone, gallocatechin, epigallocatechin, catechin, epicatechin, aromadendrin, eriodictyol, genistein, sakuranetin, naringenin, genkwanin, hispidulin and wogonin.

Positive and negative ion ESI-MS/MS analyses were performed on an Orbitrap Elite mass spectrometer (Thermo Fisher Scientific, Hemel Hempstead, UK) using a heated ESI source. The analyte solutions were diluted to 0.1 mg/mL in methanol/water (1:1) prior to analysis and were delivered using the autosampler module of an RS3000 UHPLC system (Dionex, Hemel Hempstead, UK) at a flow rate of 10 μL/min. Tandem mass spectra were recorded in CID-MS/MS mode on isolated precursor ions (2 *m*/*z* window) with collision energies ramped from 0 to 50 eV. Energies were increased by 1 eV every 12 s. The resulting runs were 10 min per sample. A full scan (50–500 *m*/*z*) was obtained then the selected precursor ion was automatically selected in data dependent mode, isolated and underwent CID-MS/MS using dry N_2_ as the collision gas. For all experiments, the acquisition time was 0.25 min per scan using FTMS mode at a resolution of 240,000. Two microscans were summed with a maximum ion accumulation time of 200 ms.

## 4. Conclusions

This study systematically investigates the fragmentation of 30 flavonoids using ESI (positive and negative ion mode) high-resolution accurate mass MS/MS. Common losses are summarised and compiled. Neutral losses such as H_2_O and CO are dominating. Radical loss of CH_3_· is consistently observed for flavonoids having methoxy groups in their structures in both positive and negative ion modes. In addition, in the positive ion mode, three key diagnostic product ions are identified: *m*/*z* 153, indicative of two OH groups on ring A; *m*/*z* 167, indicative of one OH and one methoxy group on ring A; and *m*/*z* 151, a flavanol, with no ketone oxygen but two OH groups on ring A. These findings could provide essential insight into the structural elucidation of unknown flavonoids and flavonoid metabolites.

The addition of energy breakdown graphs to the conventional tandem mass spectra dataset has proven to be a powerful analytical tool in isomer differentiation in the case of flavonoid studies. Three pairs of structural isomers are selected for detailed discussion using their MS/MS spectra and energy breakdown graphs to help visualise the proposed fragmentation pathways. It is shown that, although they may share the same common losses, ring C cleavages are often structurally indicative due to their distinct A and B ring structures and substitution patterns. Isomeric flavonoids can display distinctive patterns in their energy breakdown graphs depending on functional group locations on ring A and B.

During the investigation of rhamnetin and isorhamnetin in the negative ion mode, both flavonols exhibit a highly unusual loss of methane apart from the usual radical loss of CH_3_·. This interesting finding initiates a more detailed examination on their MS/MS spectra and energy breakdown graphs. It is concluded that the loss of methane and radical loss of CH_3_· are in competition. By interpreting the proposed mechanisms for rhamnetin and isorhamnetin, the nature of this competition is revealed for the first time, and the observation of differences in peak intensities for these two routes could be rationalised.

The work carried out in this paper provides fundamental understanding and additional information to the existing flavonoid MS literature. The results presented will aid other flavonoid related research in many disciplines, including food quality control, pharmaceutical development and drug discovery. The combination of tandem mass spectrometry and energy breakdown graphs could also be applied to studies of other natural polyphenols, especially for the differentiation of isomers, structural elucidation and qualitative studies of these complex compounds.

## Figures and Tables

**Table 1:** Identification of some of the most common neutral losses observed for positive ion ESI-MS/MS analysis of the 30 flavonoids studied.

Common Loss	Mass	Common Loss	Mass	Common Loss	Mass	Common Loss	Mass
CH_3_·	15	CH_4_O	32	CO_2_	44	CH_4_O + CO	60
H_2_O	18	2 × H_2_O	36	H_2_O + CO	46	H_2_O + 2 × CO	74
CO	28	C_2_H_2_O	42	2 × CO	56	3 × CO	84

**Table 2:** The three key diagnostic product ions, with the flavonoids (and their classes) where they were observed. The flavonoids are coloured by class: purple: flavonol; blue: flavanone; green: isoflavone; brown: flavone; red: flavanol; and grey: flavanolol.

Product Ion	Name	Nomenclature	Class
*m*/*z* 153	Quercetin	rC^1,4^_A_	Flavonol
	Morin		
	Isorhamnetin		
	Kaempferide		
	Kaempferol		
	Galangin		
	Apigenin		Flavone
	Luteolin		
	Chrysin		
	Aromadedrin		Flavanolol
	Eriodictyol		Flavanone
	Naringenin		
	Genistein		Isoflavone
*m*/*z* 167	Rhamnetin	rC^1,4^_A_	Flavonol
	Sakuranetin		Flavanone
	Genkwanin		Flavone
*m*/*z* 151	Gallocatechin	rC^3,10^_A_	Flavanols
	Epigallocatechin		
	Catechin		
	Epicatechin		

**Table 3:** The production of the three key diagnostic product ions *m*/*z* 153, 167 and 151 exemplified in the MS/MS analyses of quercetin, sakuranetin and epigallocatechin. These product ions provide direct insight into the structure of the protonated PIs of these flavonoids.

Quercetin	Sakuranetin	Epigallocatechin
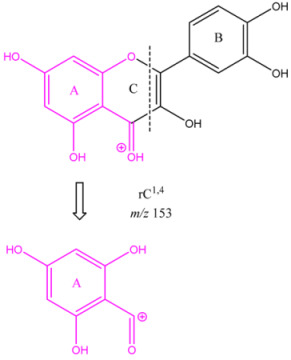	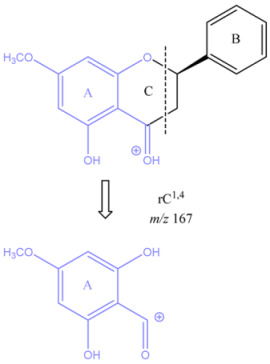	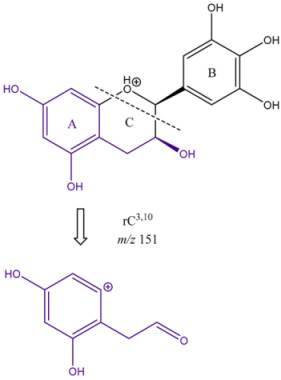

**Table 4:** Identification of some of the most common neutral losses observed for negative ion mode ESI-MS/MS analysis of the 30 flavonoids studied.

Common Loss	Mass	Common Loss	Mass	Common Loss	Mass
CH_3_·	15	C_2_H_2_O	42	CO_2_ + H_2_O	62
CH_4_	16	C_2_O	44	H_2_O + 2CO	74
H_2_O	18	H_2_O + CO	46	3 × CO	84
CO	28	2 × CO	56		

## Data Availability

The raw data are available from the University of Bristol data repository, data.bris, at https://doi.org/10.5523/bris.1mt60eklqgii41zvvz1poadro6.

## Appendix A

**Table A1:** List of abbreviations used throughout this paper.

Abbrevaition	Meaning
CI	Chemical ionisation
EBG	Energy breakdown graph
EI	Electron ionisation
ESI	Electrosprayionisation
FAB	Fast atom bombardment
HPLC	High-performance liquid chromatography
m/z	Mass-to-charge ratio
MS	Mass spectrometry
MS/MS	Tandem mass spectrometry
PI	Precursor ion
ppm	Parts per million
RDA	Retro Diels–Alder reaction
UV	Ultraviolet

**Table A2:** List of the flavonoids studied in positive ion mode, their protonated molecular precursor ion *m*/*z* and common neutral losses observed. The flavonoids are coloured by class: purple: flavonol; blue: flavanone; green: isoflavone; brown: flavone; red: flavanol; and grey: flavanolol. The red and blue shading provide a unique colour for each neutral loosed as well as providing a visual representation of simple neutral losses (blue) and losses due to RDA ring cleavages (red).

Name	Precursor *m*/*z*	Common Losses																
Rhamnetin	317	15	18	28	30	46	56	74	138	150	166	178	180					
Isorhamnetin	317	15	32	43	60	164	178											
Gallocatechin	307	18	156	168														
Epigallocatechin	307	18	156	168														
Quercetin	303	18	28	45	46	56	74	102	108	132	138	154	166	182				
Morin	303	18	28	42	46	56	70	74	138	150	154	148	176					
Kaempferide	301	15	28	32	42	56	60	136	140	148	162	151						
Diosmetin	301	15																
Hispidulin	301	15																
Catechin	291	18	126	140	144	152	168											
Epicatechin	291	18	126	140	144	152	168											
Aromadedrin	289	18	46	94	136													
Eriodictyol	289	18	110	125	126	136												
Kaempferol	287	18	28	46	56	74	110	122	134									
Fisetin	287	18	28	46	56	74	102	138	150	166								
Luteolin	287	18	42	46	108	126	134	152										
Scutellarein	287	18	28	46	110	118	168											
Sakuranetin	287	119	120	140														
Genkwanin	285	15	30	43	118													
Wogonin	285	15																
Naringenin	273	18	28	36	42	46	60	66	72	84	102	110	120	126	150	154	166	182
Genistein	271	18	28	56	112	118	122	126										
Galangin	271	18	28	42	46	56	74	90	106	118	149							
Baicalein	271	18	18	18	28	46	102	148										
Apigenin	271	42	46	118	126	152												
Chrysin	255	42	46	68	102	108	126											
Daidzein	255	18	28	56	92	110	118											
6-Hydroxyflavanone	241	18	28	42	46	78	83	104	110									
7-Hydroxyflavonone	241	15	18	28	42	46	70	78	86	104	110							
5-Hydroxyflavone	239	44	62	88	102	106	150											

**Table A3:** List of the flavonoids studied in negative ion mode, their deprotonated molecular precursor ion *m*/*z* and common neutral losses observed. The flavonoids are coloured by class: purple: flavonol; blue: flavanone; green: isoflavone; brown: flavone; red: flavanol; and grey: flavanolol. The red and blue shading provide a unique colour for each neutral loosed as well as providing a visual representation of simple neutral losses (blue) and losses due to RDA ring cleavages (red).

Name	Precursor *m*/*z*	Common Losses													
Quercetin	301	28	44	62	72	84	108	122	150	180	194				
Morin	301	18	28	44	62	72	114	126	138	150	152	154	176	194	
Rhamnetin	315	15	16	28	44	122	150								
Isorhamnetin	315	15	16	30	44	72	150	164							
Kaempferide	299	15	44												
Kaempferol	285	28	30	42	44	46	56	71	72	98	116	134	178		
Fisetin	285	28	122	150											
Galangin	269	28	42	44	46	56	68	72	86	100	116	126			
Genkwanin	283	15													
Hispidulin	299	15													
Wogonin	283	15													
Diosmetin	299	15													
5-Hydroxyflavone	237	28	42	54	66										
Baicalein	269	18	28	44	46	72	74	100							
Apigenin	269	42	44	68	72	86	88	118	120						
Luteolin	285	18	29	42	44	62	68	72	78	86	110	134	152		
Scutellarein	285	18	28	44	46	56	62	68	72	74	86	100	108	120	148
Chrysin	253	42	44	68	72	88	102	110	146						
Gallocatechin	305	18	44	58	62	84	86	102	114	126	138	140	166	168	180
Epigallocatechin	305	18	44	58	62	84	86	102	114	126	138	140	166	168	180
Catechin	289	44	84	86	110										
Epicatechin	289	44	84	86	110										
Aromadedrin	287	28	44												
Daidzein	253	18	28	42	44	56	72	93	118	120					
Genistein	269	28	42	44	56	68	72	86	88	100	110	136			
Eriodictyol	287	136													
6-Hydroxyflavanone	239	18	27	28	42	44	78	104							
7-Hydroxyflavonone	239	42	91	104	130										
Sakuranetin	285	94	120	166	192										
Naringenin	271	94	120	146	152	164	178								

## Appendix B

**Table A4:** Product ions observed in the positive ion CID-MS/MS of rhamnetin (Figure 2, Scheme 1), including observed and theoretical *m*/*z*, formulae, mass measurement error (ppm) and identity.

Observed *m*/*z*	Theoretical *m*/*z*	Formula	Error (ppm)	Identity
317.0665	317.0656	C_16_H_13_O_7_^+^	2.8	[M+H]^+^
302.0428	302.0421	C_15_H_10_O_7_^+^·	2.3	317 –CH_3_·
299.0558	299.0550	C_16_H_11_O_6_^+^	2.7	317 –H_2_O
289.0716	289.0707	C_15_H_13_O_6_^+^	3.1	317 –CO
288.0639	288.0634	C_15_H_12_O_6_^+^	1.7	317 –OCH_3_·
271.0608	271.0601	C_15_H_11_O_5_^+^	3.3	289 –H_2_O
261.0764	261.0757	C_14_H_13_O_5_^+^	2.7	289 –CO
243.0657	243.0652	C_14_H_11_O_4_^+^	2.1	271 –CO
179.0344	179.0339	C_9_H_7_O_4_^+^	2.8	299 rC^1,3^_A_
167.0343	167.0339	C_8_H_7_O_4_^+^	2.4	317 rC^1,4^_A_
151.0393	151.0390	C_8_H_7_O_3_^+^	2.0	179 –CO
149.0237	149.0233	C_8_H_5_O_3_^+^	2.7	179 –H_2_CO
139.0392	139.0390	C_7_H_7_O_3_^+^	1.4	167 –CO
137.0236	137.0233	C_7_H_5_O_3_^+^	2.2	167 –H_2_CO

**Table A5:** Product ions observed in the positive ion CID-MS/MS of isorhamnetin (Figure 2, Scheme 2), including observed and theoretical *m*/*z*, formulae, mass measurement error (ppm) and identity.

Observed *m*/*z*	Theoretical *m*/*z*	Formula	Error (ppm)	Identity
317.0664	317.0656	C_16_H_13_O_7_^+^	2.5	[M+H]^+^
302.0429	302.0421	C_15_H_10_O_7_^+^·	2.6	317 –CH_3_·
285.0402	285.0394	C_15_H_9_O_6_^+^	2.8	317 –CH_3_OH
274.0480	274.0472	C_14_H_10_O_6_^+^	2.9	302 –CO
261.0765	261.0757	C_14_H_13_O_5_^+^	3.1	?
257.0450	257.0444	C_14_H_9_O_5_^+^	2.3	285 –CO
165.0187	165.0182	C_8_H_5_O_4_^+^	3.0	?
153.0186	153.0182	C_7_H_5_O_4_^+^	2.6	317 rC^1,4^_A_
139.0393	139.0390	C_7_H_7_O_3_^+^	2.2	317 rC^4,10^_A_

**Table A6:** Product ions observed in the positive ion CID-MS/MS of kaempferol (Figure 3, Scheme 3), including observed and theoretical *m*/*z*, formulae, mass measurement error (ppm) and identity.

Observed *m*/*z*	Theoretical *m*/*z*	Formula	Error (ppm)	Identity
287.0552	287.0550	C_15_H_11_O_6_^+^	0.7	[M+H]^+^
269.0447	269.0444	C_15_H_9_O_5_^+^	1.1	287 –H_2_O
259.0603	259.0601	C_14_H_11_O_5_^+^	0.8	287 –CO
241.0497	241.0495	C_14_H_9_O_4_^+^	0.8	269 –CO/ 259 –H_2_O
231.0652	231.0652	C_13_H_11_O_4_^+^	0.0	259 –CO
213.0547	213.0546	C_13_H_9_O_3_^+^	0.5	241 –CO/ 231 –H_2_O
177.0185	177.0182	C_9_H_5_O_4_^+^	1.7	287 rC^5,10^_B_
165.0183	165.0182	C_8_H_5_O_4_^+^	0.6	287 rC^3,10^_A_
153.0184	153.0182	C_7_H_5_O_4_^+^	1.3	287 rC^1,4^_A_
133.0284	133.0284	C_8_H_5_O_2_^+^	0.0	?
121.0283	121.0284	C_7_H_5_O_2_^+^	0.8	?

**Table A7:** Product ions observed in the positive ion CID-MS/MS of fisetin (Figure 3, Scheme 4), including observed and theoretical *m*/*z*, formulae, mass measurement error (ppm) and identity.

Observed *m*/*z*	Theoretical *m*/*z*	Formula	Error (ppm)	Identity
287.0551	287.0550	C_15_H_11_O_6_^+^	0.3	[M+H]^+^
269.0446	269.0444	C_15_H_9_O_5_^+^	0.7	287 –H_2_O
259.0602	259.0601	C_14_H_11_O_5_^+^	0.4	287 –CO
241.0496	241.0495	C_14_H_9_O_4_^+^	0.4	287 –H_2_CO_2_/269 –CO/ 259 –H_2_O
231.0652	231.0652	C_13_H_11_O_4_^+^	0.0	259 –CO
213.0547	213.0546	C_13_H_9_O_3_^+^	0.5	241 –CO
185.0598	185.0597	C_12_H_9_O_2_^+^	0.5	213 –CO
149.0234	149.0233	C_8_H_5_O_3_^+^	0.7	287 rC^1,4^_B_/259 –C_6_H_6_O_2_
137.0234	137.0233	C_7_H_5_O_3_^+^	0.7	287 rC^1,4^_B_
121.0284	121.0284	C_7_H_5_O_2_^+^	0.0	149 –CO

**Table A8:** Product ions observed in the positive ion CID-MS/MS of chrysin (Figure 4, Scheme 5), including observed and theoretical *m*/*z*, formulae, mass measurement error (ppm) and identity.

Observed *m*/*z*	Theoretical *m*/*z*	Formula	Error (ppm)	Identity
255.0655	255.0652	C_15_H_11_O_4_^+^	1.2	[M+H]^+^
237.0545	237.0546	C_15_H_9_O_3_^+^	0.4	255 –H_2_O
231.0651	231.0652	C_13_H_11_O_4_^+^	0.4	255 –C_2_
227.0704	227.0703	C_14_H_11_O_3_^+^	0.4	255 –CO
213.0546	213.0546	C_13_H_9_O_3_^+^	0.0	255 –CH_2_CO
209.0596	209.0597	C_14_H_9_O_2_^+^	0.5	237 –CO
187.0754	187.0754	C_12_H_11_O_2_^+^	0.0	255 –C_3_O_2_
153.0183	153.0182	C_7_H_5_O_4_^+^	0.7	255 rC^1,4^_A_
147.0440	147.0441	C_9_H_7_O_2_^+^	0.7	255 rC^5,10^_B_
129.0334	129.0335	C_9_H_5_O^+^	0.8	147 –H_2_O

**Table A9:** Product ions observed in the positive ion CID-MS/MS of daidzein (Figure 4, Scheme 6), including observed and theoretical *m*/*z*, formulae, mass measurement error (ppm) and identity.

Observed *m*/*z*	Theoretical *m*/*z*	Formula	Error (ppm)	Identity
255.0653	255.0652	C_15_H_11_O_4_^+^	0.4	[M+H]^+^
237.0548	237.0546	C_15_H_9_O_3_^+^	0.8	255 –H_2_O
227.0704	227.0703	C_14_H_11_O_3_^+^	0.4	255 –CO
199.0755	199.0754	C_13_H_11_O_2_^+^	0.5	227 –CO
145.0285	145.0284	C_9_H_5_O_2_^+^	0.7	237 –C_6_H_4_O
137.0234	137.0233	C_7_H_5_O_3_^+^	0.7	255 rC^1,4^_A_

**Table A10:** Product ions observed in the negative ion CID-MS/MS of rhamnetin (Figure 5, Scheme 7), including observed and theoretical *m*/*z*, formulae, mass measurement error (ppm) and identity.

Observed *m*/*z*	Theoretical *m*/*z*	Formula	Error (ppm)	Identity
315.0499	315.0510	C_16_H_11_O_7_^−^	3.5	[M–H]^−^
300.0263	300.0276	C_15_H_8_O_7_^−^	4.3	315 –CH_3_·
299.0188	299.0197	C_15_H_7_O_7_^−^	3.0	315 –CH_4_
287.0552	287.0561	C_13_H_11_O_2_^−^	3.1	315 –CO
271.0240	271.0248	C_14_H_7_O_6_^−^	3.0	299 –CO
207.0292	207.0299	C_10_H_7_O_5_^−^	3.4	315 –C_6_H_4_O_2_
193.0136	193.0142	C_9_H_5_O_5_^−^	3.1	from 299
165.0189	165.0193	C_8_H_5_O_4_^−^	2.4	from 299
121.0294	121.0295	C_7_H_5_O_2_^−^	0.8	from 207?

**Table A11:** Product ions observed in the negative ion CID-MS/MS of isorhamnetin (Figure 5, Scheme 8), including observed and theoretical *m*/*z*, formulae, mass measurement error (ppm) and identity.

Observed *m*/*z*	Theoretical *m*/*z*	Formula	Error (ppm)	Identity
315.0499	315.0510	C_16_H_11_O_7_^−^	3.5	[M–H]^−^
300.0266	300.0276	C_15_H_8_O_7_^−^	3.3	315 –CH_3_·
299.0191	299.0197	C_15_H_7_O_7_^−^	2.0	315 –CH_4_
285.0394	285.0405	C_15_H_9_O_6_^−^	3.9	315 –CH_3_OH
